# The Korean herbal formulation Yukmijihwangtang stimulates longitudinal bone growth in animal models

**DOI:** 10.1186/s12906-017-1651-1

**Published:** 2017-05-02

**Authors:** Sung-Min Cho, Sun Haeng Lee, Donghun Lee, Ji Hong Lee, Gyu Tae Chang, Hocheol Kim, Jin Yong Lee

**Affiliations:** 1 Department of Clinical Korean Medicine, Graduate School, Kyung Hee University, Seoul, 02447 Republic of Korea; 2 KHI Oriental Clinic for Children, Gyeonggi, 18455 Republic of Korea; 3 Department of Pediatrics of Korean Medicine, Kyung Hee University Korean Medicine Hospital, Kyung Hee University Medical Center, Seoul, 02447 Republic of Korea; 4 Department of Herbal Pharmacology, College of Korean Medicine, Kyung Hee University, Seoul, 02447 Republic of Korea; 5 Department of Pediatrics of Korean Medicine, Kyung Hee University Hospital at Gangdong, Seoul, 05278 Republic of Korea

**Keywords:** Yukmijihwangtang, Growth plate, Longitudinal bone growth, Recombinant human growth hormone

## Abstract

**Background:**

Yukmijihwangtang (YJT) is a traditional Korean medicine that has been used to treat kidney-yin deficiency symptoms such as dizziness and tinnitus. In addition, because it is also thought to nourish kidney-yin, it has been used to treat short stature from congenital deficiency. This study evaluated the effects of YJT on longitudinal bone growth in rats.

**Methods:**

Female adolescent rats were randomly assigned to groups that received distilled water (per os [p.o.] twice a day; control), recombinant human growth hormone (rhGH; 20 μg/kg, subcutaneous [s.c.] once a day), or two different doses of YJT (100 or 300 mg/kg, p.o. twice a day). In each group, treatment was maintained for 4 days. Rats were injected intraperitoneally with 5-bromo-2’-deoxyuridine (BrdU; 50 mg/kg) to label proliferating chondrocytes on days 2 – 4. Tetracycline hydrochloride (20 mg/kg) was injected intraperitoneally to form fluorescent bands on the growth plates on day 3 for measuring the longitudinal bone growth rate. Expression of insulin-like growth factor-1 (IGF-1) and bone morphogenetic protein-2 (BMP-2) in the growth plate was identified using immunohistochemistry.

**Results:**

There was a significant increase in the rate of bone growth in the 300 mg/kg YJT group (523.8 ± 23.7 μm/day; *P* < 0.05) compared to the control group (498.0 ± 23.8 μm/day), while the 100 mg/kg YJT group exhibited a non-significant increase. The number of BrdU-positive cells in the chondrocytes of the rhGH-treated group exhibited a significant increase (103.8 ± 34.2 cells/mm^2^) compared to that of the control group (70.3 ± 19.7 cells/mm^2^), while the 300 mg/kg YJT group had a non-significant increase. Additionally, IGF-1 and BMP-2 were highly expressed in the growth plate in the 300 mg/kg YJT and rhGH groups.

**Conclusions:**

YJT increased the longitudinal bone growth rate by stimulating chondrocyte proliferation with increasing increments of local IGF-1 and BMP-2 expression. Based on these findings, YJT may be a therapeutic candidate for the treatment of growth retardation during adolescence.

## Background

Yukmijihwangtang (YJT) contains six medicinal herbs: *Rehmannia glutinosa, Cornus officinalis, Dioscorea batatas*, *Alisma orientale, Poria cocos,* and *Paeonia suffruticosa.* YJT has previously been prescribed to treat cancer, dementia, diabetes, hypertension, menopausal syndromes, nephritis, neurasthenia, neurosis, Parkinson’s disease, systemic lupus erythematosus, and thrombocytopenic purpura. YJT possesses anti-aging, anti-carcinogenic, anti-hypertensive, anti-inflammatory, anti-osteoporotic, anti-oxidant and neuromodulating pharmacological effects. Therefore, administration of YJT is thought to delay declines in learning and memory, diminish the aging process and geriatric diseases, and strengthen estrogenic activity during menopause. Additionally, YJT has been used to treat clinical kidney-yin deficiency symptoms such as dizziness, hectic fever, dry mouth and throat, night sweats, spermatorrhea, excessive thirst and liquid intake, red tongue with less coating, and rapid pulse [1]. Previous experimental studies have shown that YJT enhances immune function by regulating the secretion of neurotransmitters and hormones associated with the hypothalamus-pituitary-adrenal axis [2], as well as strengthening bone by stimulating the remodeling process [3]. YJT also increases cognitive function via improvements in synaptic plasticity [4] and protects against neurodegeneration by adjusting mitochondrial function and downregulating apoptosis signals [5]. In a model of obesity, YJT improved energy metabolism and sensitivity to insulin and leptin, which, in turn, inhibited weight gain and visceral fat accumulation [6, 7]. Additionally, YJT decreased prostate enlargement in a model of prostatic hyperplasia [8]. A previous clinical study found that YJT effectively improves hyperglycemia and renal function, while adverse events from the treatment were infrequent or mild [9].

Many herbal medicines have been evaluated to determine whether they promote skeletal growth. For example, Bojungikki-tang [10], Cheunggyeongsamul-tang [11], and Jaoga-yukmiwon [12] increased bone growth. It is expected that YJT will also promote bone growth, as the method of nourishing the kidney-yin has been used clinically on short statured patients with congenital deficiencies in Korea [13], and this compound is similar to the growth-promoting Jaoga-yukmiwon medicine; furthermore, YJT in conjunction with Zi He Che (Hominis placenta) suppresses bone resorption [14]. However, non-significant differences in X-ray measurements of femur and tibia length following YJT treatment were observed previously, even though YJT increased serum levels of growth hormone (GH) and thyroid hormones compared with the control group [15].

Due to the contradictory reports regarding the effect of YJT on bone growth, the present study evaluated the effects of two different concentrations of YJT in adolescent female rats. YJT doses of 100 and 300 mg/kg were used because these concentrations had significant effects on bone growth compared to a control group in a previous experiment that investigated adolescent female rats [10]. These two concentrations were less than a daily dose of 2.4 g/d, which was converted from the recommended clinical daily dose of 4.5 g/d in humans [16], and were expected to have few side effects. Additionally, one group of rats in the present study received GH as a positive control, because GH affects final height after birth and plays a variety of roles during the growth spurt in adolescence [17]; furthermore, over-expression of the GH gene in rats significantly increases the growth of the body and organs [18]. However, it has also been shown that administration of GH to prepubertal rats promotes tibial growth and weight increases (in females but not in males) [19]. Therefore, adolescent female rats were used as the experimental animals in the present study and tibial growth was assessed because height is more related to the lower extremities than the upper extremities [20]. Furthermore, with respect to the lower limbs, tibia length is the best indicator of height [21].

In this study, longitudinal bone growth was determined by evaluating increases in the length of the tibial growth plate over 48 h. To accomplish this, the number of 5-bromo-2’-deoxyuridine (BrdU)-positive cells in the growth plate was determined to verify cartilage proliferation, and immunohistochemistry was applied to assess the expression of bone morphogenetic protein-2 (BMP-2) and insulin-like growth factor-1 (IGF-1), which are involved in bone growth in the growth plate [22].

## Methods

### Sample preparation

Radix Rehmanniae Prepara, Corni Fructus, Dioscoreae Rhizoma, Alismatis Rhizoma, Poria, and Moutan Cortex were purchased from Yaksoodang (Seoul, Korea). They were identified by Professor Hocheol Kim and voucher specimens (Nos. 14032809, 14032807, 14032808, 14032812, 14032806, and 14032805, respectively) were deposited in the Department of Herbal Pharmacology (College of Korean Medicine) of Kyung Hee University (Seoul, Korea).

The prepared root of *R. glutinosa,* fruit of *C. officinalis,* rhizomes of *D. batatas* and *A. orientale,* scleorotia of *P. cocos,* and root bark of *P. suffruticosa* were mixed at a ratio of 4:2:2:1:1:1, which is similar to the ratio (8:4:4:3:3:3) described in Qian Yi’s ancient book “Key to Therapeutics of Children’s Disease” [23], and was extracted with distilled water twice for 4 h at 100 °C in a reflux apparatus (Table 1). The extracts were filtrated and concentrated under reduced pressure and the samples were lyophilized to yield powders; the yield of the extract was 72.7%.

**Table 1:** Composition of Yukmijihwangtang

Herbal name	Plant name	Plant part utilized	Amount (g)
Radix Rehmanniae Prepara	*Rehmannia glutinosa*	Prepared root	36.4
Corni Fructus	*Cornus officinalis*	Fruit	18.2
Dioscoreae Rhizoma	*Dioscorea batatas*	Rhizomes	18.2
Alismatis Rhizoma	*Alisma orientale*	Rhizomes	9.1
Poria	*Poria cocos*	Scleorotia	9.1
Moutan Cortex	*Paeonia suffruticosa*	Root bark	9.1

**Fig. 1:**
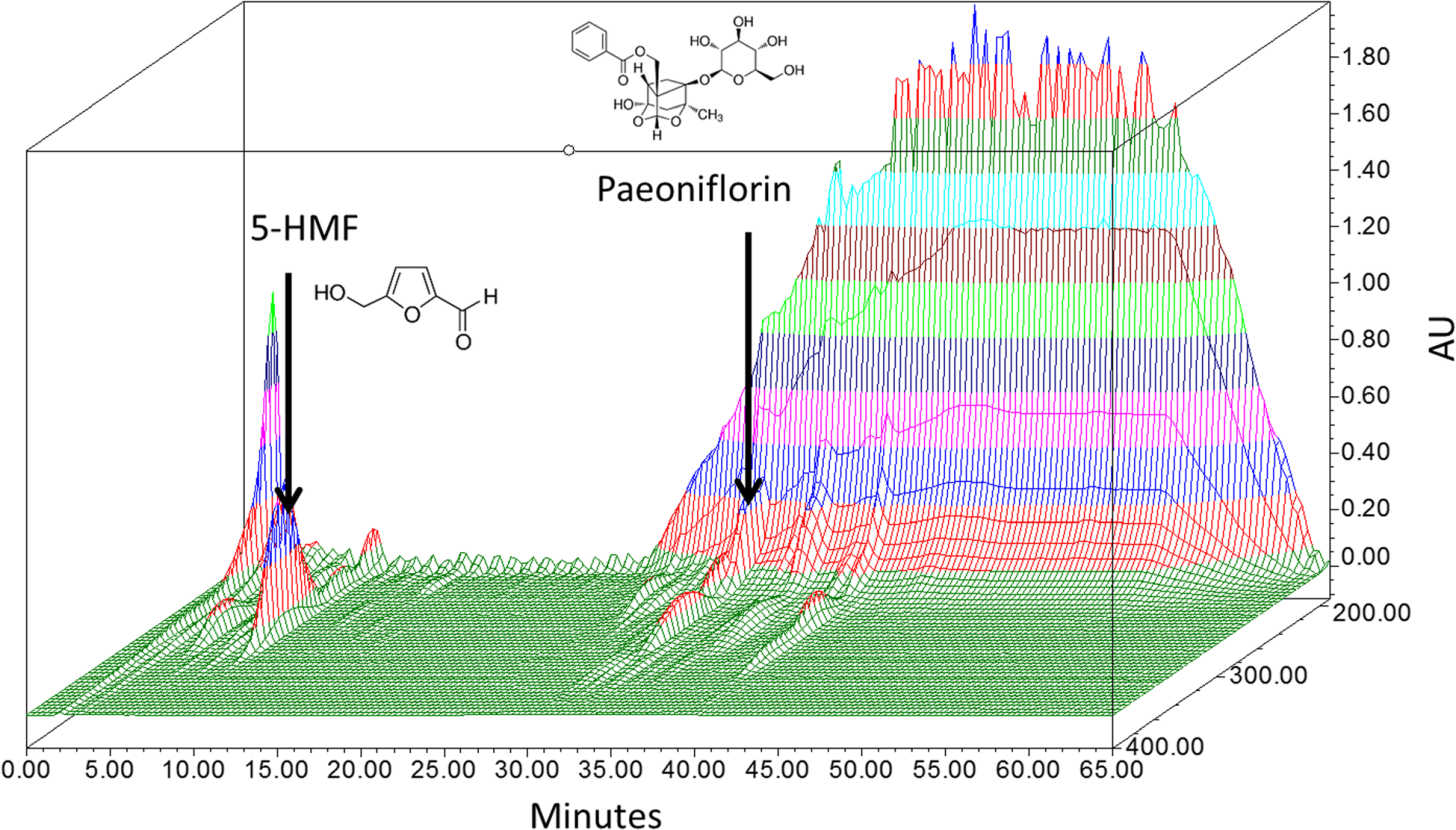
Three-dimensional high-performance chromatography of Yukmijihwangtang (YJT)

The quantitative authentication of YJT was performed on Waters instrument (Milford, MA, USA) equipped with a Waters 1525 pump, Waters 2707 autosampler, and a Waters 2998 PDA detector using a Sunfire™ Octadecyl silyl silica C18 column (particle size, 5 μm; 250 × 4.6 mm). The column was equilibrated with distilled water (solvent A) and 50% MeOH (solvent B) at a flow rate of 1.0 mL/min. The column was eluted as follows: 0–30 min, 20% solvent B; 31–40 min, 60% solvent B; 41–55 min, 75% solvent B; 55–60 min, 60% solvent B; 60–65 min, 20% solvent B. The high-performance liquid chromatogram of YJT is shown in Fig. 1. YJT contained two representative components: 8.71 mg/mL of 5-hydroxymethyl-2-furaldahyde for *R. glutinosa* and 4.19 mg/mL of paeoniflorin for *P. suffruticosa*.

### Animals and administration

This study included 32 female Sprague-Dawley rats (4-weeks-old) that weighed 70 ± 10 g each (Samtako Co., Osan, Korea). All experimental procedures were performed in accordance with the animal care guidelines of the Kyung Hee University’s Institutional Animal Care and Use Committee (KHUASP (SE)-10-034). The 3-weeks-old animals divided by four body weight groups (30–40 g, 40–50 g, 50–60 g, and 60–70 g). They were randomly put in the eight cages (4 rats/cage) by way of uniform weight distribution and the four treatments were assigned to the randomized cages. The animals were housed under controlled conditions (temperature of 23 ± 2 °C, relative humidity of 55 ± 10%; 07:00–19:00 light cycle with food and water available ad libitum). After 5 days of acclimatization, the treatments were maintained for 4 consecutive days in each group. The control group received oral administrations of distilled water twice a day, the positive control group was subcutaneously injected with 20 μg/kg of recombinant human GH (rhGH; LG Life Science, Daejeon, Korea) once a day, and the two YJT groups received oral administrations of either 100 or 300 mg/kg, twice a day. Oral administration occurred at 09:00 and 21:00, and injections were performed at 09:00. All of the administrations were done in the home cage. After treatment, the animals were sacrificed prior to analysis.

### Measurement of longitudinal bone growth

To investigate the effects of YJT on longitudinal bone growth, tetracycline was used as a fluorescence marker to label the bone line on the surface of the tibia; tetracycline is fluorescent under ultraviolet illumination. The rate of longitudinal bone growth was assessed by measuring the distance between the fluorescent line formed by tetracycline and the epiphyseal end line of the growth plate. To accomplish this, all rats received intraperitoneal (i.p.) injections of tetracycline hydrochloride (20 mg/kg; Sigma Chemicals Co., St. Louis, MO, USA) on day 3 of the experiment. On days 2–4, the rats received i.p. injections of BrdU (50 mg/kg, Sigma) to label proliferating cells, and on day 5 all animals were anesthetized with ether and sacrificed. The dissected tibias were fixed in 4% paraformaldehyde for 48 h and then underwent decalcification by immersion in a 10% ethylene diamine tetra acetic acid solution (Sigma) for 24 h. After dehydration by immersion in a 30% sucrose solution for 1 day, each bone sample was longitudinally sectioned (40 μm thick) using a sliding microtome (HM440E; Carl Zeiss, Oberkochen, Germany). The focus was placed between the epiphyseal plate and the fluorescent band that was formed by the chelation of tetracycline and calcium on the epiphyseal plate following injection of tetracycline, which was visible under a fluorescence microscope (Olympus, Tokyo, Japan). Measurements and calculations of bone growth were performed using ImageJ software (National Institutes of Health, Bethesda, MD, USA) and mean values were obtained from three different sections within the fluorescent band gap.

### Measurement of BMP-2 and IGF-1 in the growth plate

The tissue sections were washed twice in 0.1 M phosphate-buffered saline (PBS), twice in 1% triton X-100 (Sigma) for 15 min, and then twice with 0.5% bovine serum albumin (BSA; Sigma) dissolved in PBS for 15 min. The sections were then incubated with goat BMP-2 primary antibody and rabbit IGF-1 primary antibody (1:200; Santa Cruz Biotechnology, Santa Cruz, CA, USA) overnight at room temperature in a humidity chamber. After 24 h, the sections were washed twice with 0.5% BSA in PBS and then incubated with either biotinylated anti-goat secondary antibody (1:200; Vector Laboratories, Burlingame, CA, USA) or biotinylated anti-rabbit secondary antibody (1:200; Jackson Immuno Research Laboratories, West Grove, PA, USA) for 1 h. After being washed twice with PBS for 15 min, the sections were incubated with an avidin-biotin-peroxidase complex (1:100, Vectastain ABC Kit; Vector Laboratories) for 1 h at room temperature. After another wash with PBS, the sections were stained and reacted with a 0.05% 3, 3-diaminobenzidine (DAB) solution containing hydrogen peroxide in PBS. The reaction was stopped by washing them with PBS and then the slides were dehydrated with solutions of 50, 75, 95, and 100% ethanol and xylene, in that order. The sections were mounted on glass slides with Permount medium solution (Fisher Scientific, Waltham, MA, USA) and micrographs of the sections were taken.

### Statistical analysis

All data are presented as means ± standard deviation (SD). The effects of the different treatments were compared by Student’s *t*-test using GraphPad Prism 5 software (GraphPad Software Inc., San Diego, CA, USA); *P* values < 0.05 were considered to be statistically significant.

## Results

### Effects of YJT on the rate of longitudinal bone growth

**Fig. 2:**
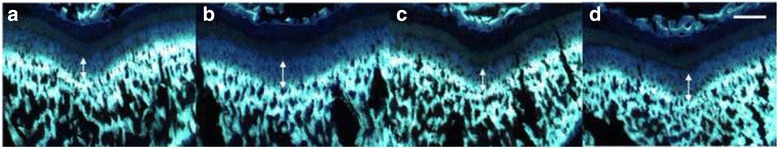
Fluorescent photomicrographs of longitudinal sections of the proximal tibia in the growth plate. **a** Control group (distilled water), **b** recombinant human growth hormone (rhGH) group (20 μg/kg), **c** 100 mg/kg YJT group, and **d** 300 mg/kg YJT group. The *fluorescent line* corresponds to the injection of tetracycline, which binds with calcium and can be detected with ultraviolet illumination. The *arrow* between the fluorescent line formed by the tetracycline and the epiphyseal end line of the growth plate indicates the extent of bone growth during the 48 h study period. Scale bar = 200 μm

**Fig. 3:**
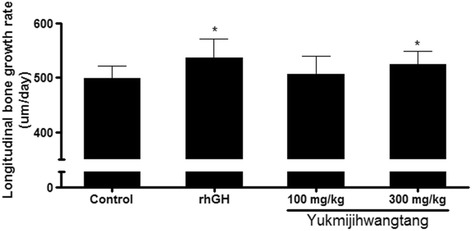
Effects of YJT on longitudinal bone growth rate in adolescent rats. The control group: received distilled water and the rhGH group received 20 μg/kg rhGH. Values are means ± SD of eight rats. *P < 0.05 compared to the control group

The effects of YJT on bone growth were assessed by taking measurements of the gap between the growth plate and the band formed by tetracycline at three different locations to obtain an average value (Fig. 2). Next, the extent of bone growth over 48 h was converted to a daily growth rate. There was a significant acceleration of longitudinal bone growth in the 300 mg/kg YJT and rhGH groups compared to the control group (Table 2, Fig. 3). However, there was no significant difference in the rate of bone growth between the 100 mg/kg YJT group and the control group.

**Table 2:** Longitudinal bone growth rate in adolescent female rats

Group	Longitudinal bone growth (μm/day)
Control	498.0 ± 23.8
Recombinant human growth hormone (20 μg/kg)	536.3 ± 34.7^*^
Yukmijihwangtang (100 mg/kg)	506.4 ± 32.9
Yukmijihwangtang (300 mg/kg)	523.8 ± 23.7^*^

The control group received distilled water. Values are means ± SD of eight rats. Statistical significance was determined with a *t*-test: ^*^ *P* < 0.05 compared to the control group

### Effects of YJT on chondrocyte proliferation

**Fig. 4:**
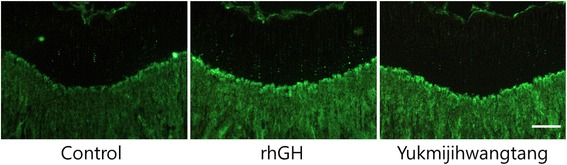
Representative images of 5-bromo-2’-deoxyuridine (BrdU)-labeled chondrocytes in the growth plates in the proximal tibia. The control group received distilled water, the rhGH group received 20 μg/kg rhGH and the YJT group received 300 mg/kg YJT. *Green*: BrdU-labeled chondrocyte. Scale bar = 100 μm

**Fig. 5:**
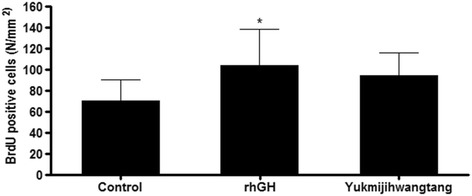
Effects of YJT on chondrocyte proliferation in the growth plates of adolescent female rats. BrdU-positive cells were quantified. The control group received distilled water, the rhGH group received 20 μg/kg rhGH and the YJT group received 300 mg/kg YJT. Values are means ± SD of eight rats. *P < 0.05 compared with the control group

BrdU-labeled cells were observed in the chondrocytes (Fig. 4). The number of BrdU-positive cells in the rhGH group was significantly higher than that in the control group, but the number of BrdU-positive cells in the 300 mg/kg YJT group did not significantly differ from that in the control group (Table 3, Fig. 5).

**Table 3:** Numbers of BrdU-positive cells in the growth plate

Group	BrdU-positive cells/mm^2^
Control	70.3 ± 19.7
Recombinant human growth hormone (20 μg/kg)	103.8 ± 34.2^*^
Yukmijihwangtang (300 mg/kg)	93.8 ± 22.0

The control group received distilled water. Values are means ± SD of eight rats. Statistical significance was determined with a *t*-test: ^*^ *P* < 0.05 compared to the control group

### Effects of YJT on the expression of BMP-2 and IGF-1

**Fig. 6:**
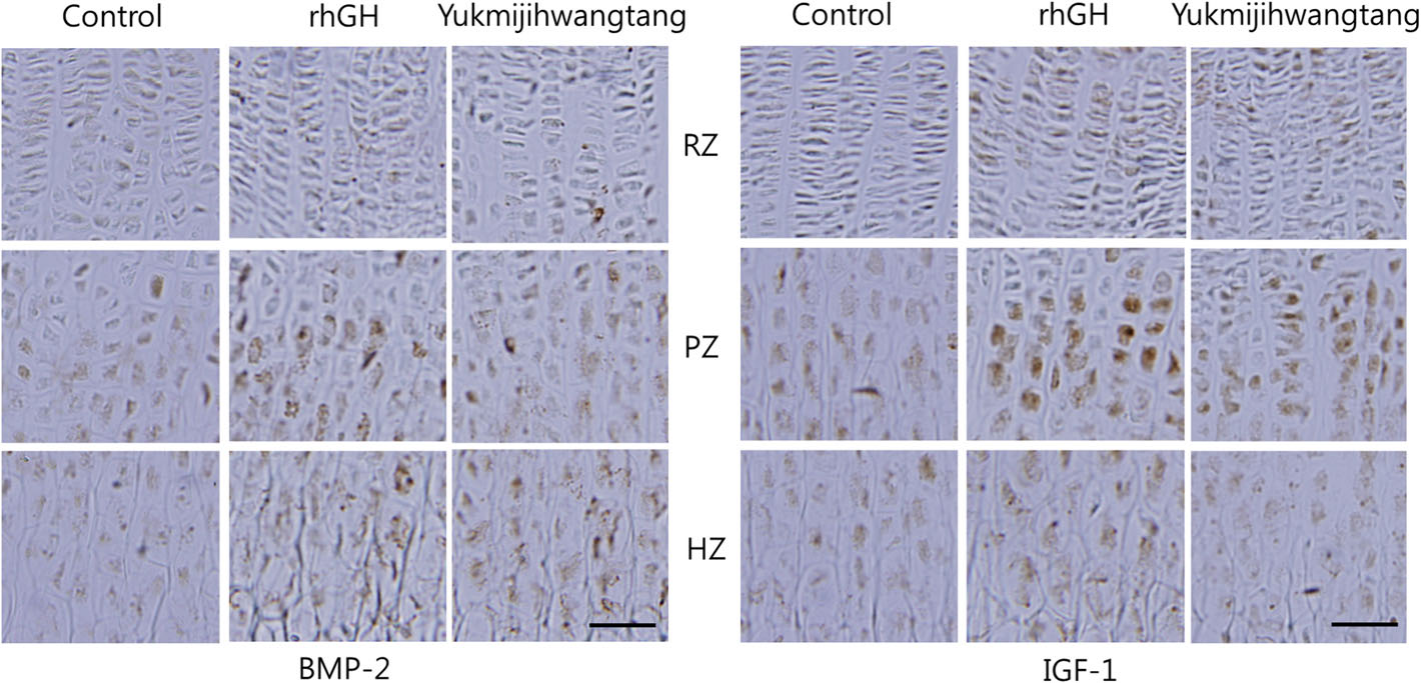
Immunohistochemical localization of bone morphogenetic protein (BMP)-2 and insulin-like growth factor (IGF)-1 in the growth plate. The control group received distilled water, the rhGH group received 20 μg/kg rhGH and the YJT group received 300 mg/kg YJT. RZ, resting zone; PZ, proliferative zone; HZ, hypertrophic zone. Scale bar = 400 μm

Immunohistochemical experiments were conducted to evaluate the expression of BMP-2 and IGF-1 in the three principle zones of the growth plate. In all groups, the largest changes in BMP-2 and IGF-1 staining were evident in the cytoplasm of the proliferative and hypertrophic zones. Treatment with 300 mg/kg YJT markedly increased the expression of BMP-2 and IGF-1 in the proliferative zone and hypertrophic zone of the growth plate compared to treatment with distilled water. Additionally, expression of BMP-2 and IGF-1 was higher in the rhGH group than in the control and YJT groups (Fig. 6).

## Discussion

In the present study, oral administration of YJT for 4 days significantly increased the rate of longitudinal bone growth and chondrocyte proliferation in the proximal tibial growth plate compared to the control group. YJT also increased the expression of IGF-1 and BMP-2 in the hypertrophic zone of the growth plate.

A study that measured changes in bone length and serum hormone levels after YJT treatment found that this herbal medicine increased the levels of GH and thyroid hormone, but did not affect bone length or body weight [15]. In the 300 mg/kg YJT group in the present study, there was an increase in the rate of bone growth of 5.2% relative to the control group. Tetracycline accumulates in newly formed bone and produces fluorescent lines subsequent to its injection. The gap between the fluorescent line and the chondro-osseous junction indexes the rate of bone growth rate [24]. Using this procedure, the present study found that a higher dose of YJT increased the rate of bone growth. However, there was a non-significant increase in bone growth rate in the 100 mg/kg YJT group, which suggests that a certain concentration of YJT is required to significantly promote longitudinal bone growth. The 10-fold dose of YJT used in a previous study [15] might be an inappropriate dose for stimulating bone growth. Further study will be necessary to identify the most effective concentration for bone growth.

The growth plate matures due to the influences of various growth factors and hormones until late adolescence. The perichondrium, which is a key factor during growth plate maturation, plays an important role in osteoblast formation and capillary penetration [25]. Additionally, perichondrium cells regulate chondrocytes via BMPs, fibroblast growth factors, and Wnt signals. BMPs belong to the transforming growth factor (TGF)-β family and act as a growth and differentiation factor [26]. They determine chondrogenesis in pro-chondrogenic cells, accelerate the condensation process and differentiation into chondroblasts and chondrocytes, and aid in the formation of bone tissue [27, 28]. BMP-2 was selected as an indicator of bone growth in the present study because TGF-β is unable to generate all types of bone tissue by itself, whereas BMP-2 alone induces ectopic bone formation [29]. BMP-2 also regulates the retinoid pathway, which causes chondrocyte proliferation [30], and promotes bone formation via the suppression of noggin, which inhibits bone tissue generation [31]. BMP-2 also accelerates bone growth by promoting chondrocyte proliferation and hypertrophy [32]. GH stimulates local-acting IGF-1 [33], which, as an essential growth-promoting polypeptide during normal bone metabolism, aids biosynthesis and substrate production in association with insulin-like anabolism [34, 35]. As a result, longitudinal bone growth is stimulated through chondrocyte proliferation and hypertrophy. IGF-1 was selected as another indicator of bone growth in the present study, because the heights of proliferative and hypertrophic zones in chondrocytes decrease under conditions of deficient IGF-1 [36].

The growth plate consists of a resting zone that contains immature cells, a proliferative zone that involves mature chondrocytes, and a hypertrophic zone that is comprised of enlarged chondrocytes [25]. The preparatory process for proliferation, in which progenitor cells are aligned in a parallel direction along the long axis of the bone, occurs in the resting zone [37]. During this process, flattened chondrocytes divide in the longitudinal direction and synthesize extracellular substrates that are essential for the cartilage matrix structure in the proliferative zone. Mineralization is accomplished by the secretion of large amounts of substrate protein, as well as by increases in the intracellular calcium concentration in the hypertrophic zone [22]. In the present study, the expression of BMP-2 and IGF-1 markedly increased in the proliferative and hypertrophic zones in the YJT groups, which suggests that YJT stimulated longitudinal bone growth by chondrocyte proliferation and hypertrophy in these areas. These results are similar to others obtained with bone growth-promoting herbal prescriptions.

Longitudinal bone growth appears to be a complex process that involves the proliferation and longitudinal hypertrophy of chondrocytes and the production of bone matrix. BrdU is a thymidine analog that bonds to S phase cells; thus, BrdU-labeled cells can be measured to confirm chondrocyte proliferation in the growth plate [38]. In the present study, the rhGH and YJT groups showed 1.5- and 1.3-fold increases in BrdU-positive cells, respectively, compared to the control group. However, while rhGH treatment resulted in a significant increase in BrdU-positive cells, YJT caused a non-significant increase. Therefore, the growth-promoting effects of YJT might be properly explained by other growth mechanisms.

Diosgenin, a steroid saponin organizing *Dioscorea*, increases bone formation by enhancing the synthesis and secretion of Type 1 collagen and alkaline phosphate. Expression of bone marker proteins, Runx2 and osteopontin, stimulated by diosgenin also enhances calcium deposits within the extracellular matrix of bone [39]. Alisol-B, a steroid from *A. orientale*, suppresses osteoclastogenesis and prevents bone loss [40]. The bone growth-promoting effect of YJT might be due to enhancement of the extracellular matrix or prevention of bone loss. Further studies are needed to investigate these potential mechanisms.

In the present study, a large concentration of YJT produced a bone growth effect, but the induced growth was still less than that induced by rhGH, which is the approved treatment for idiopathic short stature [41]. However, long-term GH treatment is expensive and the ultimate height increase may still be small if the treated child does not have a GH deficiency [42]. GH treatment is generally safe but its long-term safety has yet to be confirmed and rare adverse effects, such as edema, pseudotumor cerebri, gynecomastia, hyperinsulinemia, and hyperglycemia, have been described [43]. The present findings suggest that YJT may be an alternative treatment for short stature that does not have the high cost or potential risks associated with GH treatment.

## Conclusions

The present study demonstrated that at certain concentrations, YJT promotes longitudinal bone growth velocity via increases in the expression of BMP-2 and IGF-1 in the proliferative and hypertrophic zones of the growth plate. Although YJT has the potential to be a cost-effective and safe treatment for short stature, further studies are needed to verify the active components of this herbal medicine.

## Abbreviations

BMP: Bone morphogenetic protein
BSA: Bovine serum albumin
GH: Growth hormone
IGF: Insulin-like growth factor
PBS: Phosphate-buffered saline
rhGH: Recombinant human growth hormone
YJT: Yukmijihwangtang
